# Information needs and patient perceptions of the quality of medication information available in hospitals: a mixed method study

**DOI:** 10.1007/s11096-020-01125-x

**Published:** 2020-08-28

**Authors:** Charlotte L. Bekker, Shaghayegh Mohsenian Naghani, Stephanie Natsch, Naomi S. Wartenberg, Bart J. F. van den Bemt

**Affiliations:** 1 Department of Pharmacy, Radboud University Medical Center, Radboud Institute for Health Sciences, Geert Grooteplein Zuid 10, 6525 GA Nijmegen, The Netherlands; 2 Department of Pharmacy, Sint Maartenskliniek, Hengstdal 3, 6574 NA Nijmegen, The Netherlands; 3 Department of Clinical Pharmacy and Toxicology, Maastricht University Medical Center, P. Debyelaan 25, 6229 HX Maastricht, The Netherlands

**Keywords:** Medication information, Mixed methods, Outpatient, Patient education, Pharmaceutical care

## Abstract

*Background* Providing appropriate medication information to patients is of utmost importance for optimal pharmacotherapy. (Un)intentional miscommunication and information gaps resulting in unmet needs could negatively affect patient’s ability to use their medication properly. *Objective* To identify the information needs and patient perceptions of the quality of medication information available in hospitals in the Netherlands. *Setting* Cardiology, oncology, or rheumatology department of five hospitals. *Methods* Adult cardiology, oncology, and rheumatology patients participated in this mixed-method study. Focus groups and individual interviews were held to identify patients’ views on the medication information and their information needs. Outcomes were used to construct a questionnaire that was used in a survey among patients to compare existing medication information with patients’ needs, and to judge the quality of the provided information. *Main outcome measure* Patients needs with medication information. *Results Four themes derived from interviews with 44 patients: (1) Content; almost all patients acknowledged to receive insufficient information not meeting their personal needs. (2) Moment of delivery;* patients were dissatisfied with the timing. *(3) Method of delivery; patients highly preferred verbal and written information. (4) Contextual quality prerequisites that should be met according to patients; medication information should be* accessible, comprehensive, reliable and understandable*. A total of* 352 patients completed the questionnaire. *Almost all patients reported all items as important, whereas up to 74.6% patients were not informed. Up to half of the patients perceived verbal information from healthcare providers, written information of leaflets and folders of insufficient quality. Conclusion* Patients attending Dutch hospitals have needs for extensive medication information, which should be tailored to their individual needs. According to patients the quality of medication information available in hospitals can be improved.

**Electronic supplementary material:**

The online version of this article (10.1007/s11096-020-01125-x) contains supplementary material, which is available to authorized users.

## Impacts on practice

- Medication information should be tailored to patients’ individual needs
- Quality of medication information, in terms of accessibility, comprehensiveness, reliability and understanding, can be improved
- Healthcare providers could should pay attention to patient information needs for optimisation, which might be facilitated by improved patient-provider communication

## Introduction

Providing appropriate medication information to patients is of utmost importance for optimal pharmacotherapy, medication adherence and disease control [1, 2]. Patients with inadequate knowledge about their medication, receiving inappropriate or non-understandable information are less likely to adhere to their therapy, which could lead to inadequate medication use and decreased therapy efficacy [3, 4]. Well informed patients are also more empowered and more likely to participate in shared-decision making [5, 6].

Medication information needs of patients vary greatly and strongly depend on the diagnosis and type of disease and patient characteristics such as socioeconomic status and age [7]. Furthermore, patients’ desire for information is likely to change over time and with their experience of medication therapy [8, 9]. Information should therefore be provided in such a way that it is tailored to the individual patient over the course of treatment.

Healthcare providers and patients have different understanding of the information patients should receive about medications [10]. Patients have greater informational needs on, for instance, adverse events, than healthcare providers think they should provide. Moreover, patients are not always satisfied with the information they receive [11, 12]. Halbach et al. [13] showed that breast cancer patients had high unmet information needs, especially for patients with limited health literacy. (Un)intentional miscommunication and information gaps resulting in unmet needs could negatively affect patient’s ability to manage their medication use properly.

A recent review described the medication information relevant to patients and showed that patients particularly desired safety-related information including adverse drug reactions and drug-drug interactions [14]. However, most of the included studies described information topics desired by patients and to a lesser extent a comparison with the information that patients currently received. Furthermore, most studies were conducted in the community setting and the few studies that had assessed the needs of outpatient hospital patients used a questionnaire design that lacked in-depth information. Assessments of whether patients in the outpatient setting are sufficiently informed according to their personal needs is crucial for improving the information provision. Likely, information provision and information needs vary greatly between countries due to healthcare and cultural differences. Few studies have focussed on the outpatient setting in the Netherlands.

## Aim of the study

This study aimed therefore to identify the information needs and patient perceptions of the quality of medication information available in hospitals in the Netherlands.

## Ethics approval

The Medical Research Ethical Committee of the Radboud university medical center approved the study (file numbers 2017-3199, 2018-4406, 2018-4480 and 2018-5038). Patients gave written informed consent for participation in the interviews or focus groups and oral informed consent for participation in the survey.

## Methods

### Study design and setting

A mixed-methods study with an exploratory sequential design was used to obtain a comprehensive assessment of patients’ needs with medication information [15]. First, a qualitative data collection with in-depth patient focus groups and individual interviews was conducted. Then, quantitative data collection was carried out with a questionnaire among a larger patient sample. The study included ambulatory patients from the outpatient cardiology, oncology, or rheumatology department as these comprise generally substantial numbers of patients using long-term prescription medication. The study was conducted in 5 hospitals between May 2017 and March 2019 in the Netherlands: two large teaching hospitals, one university’s hospital, one general hospital, and one specialized hospital. Usual care for cardiology, oncology and rheumatology patients in the participating centres consisted of doctors and/or nurses providing verbal and/or written (folders) medication information during consultation when considered necessary or when the patient explicitly requested information. Disease-specific oncology and rheumatology medication was dispensed by the outpatient pharmacy, whereas cardiology patients received their disease-specific medication from the outpatient or community pharmacy. Pharmacies generally provided verbal and written medication information (leaflets).

### Study population

Adult patients aged ≥ 18 years were eligible for inclusion when being prescribed cardiovascular, anticancer, or rheumatology medication and being able to communicate in Dutch.

### Qualitative phase

#### Participants inclusion

Focus groups were held with oncology or rheumatology patients. Individual interviews were held with cardiology patients for convenient reasons related to planning and interview logistics. Eligible oncology patients were approached by their oncologist until a sufficient number of patients expressed interest in participation (i.e. up to 12 patients). Rheumatology patients were approached from a large sample of patients who had shown interested in participation in a focus group from another study and were invited for participation in this study. Furthermore, rheumatology patients were also invited through a local society for rheumatology patients by email. Patients who expressed interest received additional study information by post. Cardiology patients consecutively visiting the cardiology department were approached by a nurse, whom assessed patient’s ability to participate in an interview, and if patients agreed to participate, they were directly interviewed after their consult. Focus groups were conducted before the individual interviews.

#### Interviews

A semi-structured interview guide was developed by the research team with members experienced in hospital and outpatient pharmacy practice as well as pharmacy related research in multiple meetings. *First, focus groups were conducted using the* semi-structured interview guide. A trained moderator with a health background led the focus groups and questioned patients about: (1) views on the current medication information provision, (2) medication information needs, including content, information sources, and moment, and (3) possibilities for improvement. Thereafter, a similar interview guide was used for individual interviews with cardiology patients held by a researcher (SMN). Questions focussed on disease-specific medication. All interviews were conducted in a hospital room and field notes were made by a researcher. Participants gave written informed consent prior to participation. Interviews were audio recorded and transcribed verbatim.

#### Data analysis

Transcripts were analysed using thematic analysis with an explanatory, descriptive approach in the software programs ATLAS.ti 8.3.20 and MAXQDA 11 [16]. Firstly, relevant text fragments were identified and selected with open codes by two researchers independently (CB and SMN or CB and NW). These were compared, and disagreements were discussed until consensus was achieved. Thereafter, axial and selective coding was applied. During axial coding, open codes were placed into categories. Overarching themes were formulated during selective coding. These steps were performed by the first researcher (SMN or NW) and then critically reviewed by the second researcher (CB). Discrepancies were resolved through consensus. Outcomes were discussed with the research team. Quotes were translated by a researcher (SMN or NW) and critically reviewed by a second researcher (CB). The COREQ checklist was used to ensure comprehensive reporting [17].

### Quantitative phase

#### Participants inclusion

A survey with a self-administered paper questionnaire was conducted among cardiology, oncology, and rheumatology patients. Consecutive oncology and rheumatology patients were approached to fill in the questionnaire when visiting the outpatient pharmacy’s department by a pharmacy technician and cardiology patients when visiting the outpatient cardiology department by a researcher. The questionnaire could directly be filled in while waiting or at home and sent back by post (free of charge).

#### Questionnaire

Identified themes derived from the qualitative phase were used to construct a questionnaire to further assess patients’ needs among a larger patient sample in comparison with the current medication information provision. Items that reflected patients’ needs in the interviews were formulated as statements. Items that had the same content as the statements of the Dutch Satisfaction with Information about Medicines Scale (SIMS) were similarly formulated (see Appendix for Dutch questionnaire) [18]. Most content of the questionnaire was similar for each disease, but some disease specific items, e.g. those mentioned only by cardiology patients, were generated. Questions focussed on disease-specific medication. Participants were asked to report: (1) socio-demographic characteristics, including gender, age, and type of disorder, (2) whether they had been informed about items using “yes/no/not applicable”, (3) needs per item using a five-point Likert scale with 1 = “unimportant”, 2 = “slightly unimportant”, 3 = “neutral”, 4 = “slightly important”, and 5 = “important”, (4) from whom they received information and what they preferred, and (5) judgement of quality prerequisites of medication information (accessible, comprehensive, reliable, and understandable) using a five-point Likert scale with 1 = “very bad”, 2 = “bad”, 3 = moderate, 4 = “good”, and 5 = “very good” (“Appendix 2”). The questionnaire was piloted with cardiology patients on interpretation.

#### Data analysis

Data were descriptively analysed using the Statistical Package for Social Sciences (SPSS) version 25. Categorical data were reported as percentages and continuous data as means with standard deviations (SD). Outcomes per statement on the Likert-scales were presented as medians with interquartile ranges (pp. 25–75). Quality aspect outcomes “very bad to moderate” were categorised as “insufficient”.

## Results

### Qualitative phase

In total, 44 patients participated in the qualitative research phase (mean [SD] 64.5 [12.3] years, 38.6% male); 20 individual interviews with cardiology patients that lasted between 10 and 36 min, one focus group with 9 oncology patients, and two focus groups with 15 rheumatology patients, which lasted around two hours. Four main themes of information need of patients were identified: (1) content of medication information, (2) moment of delivery, (3) method of delivery, and 4) contextual quality prerequisites (Table 1).

**Table 1:** Four themes derived from the qualitative data

Content	Moment of delivery	Method of delivery	Quality prerequisites
Insufficient information is provided	Dissatisfied with quantity and timing of information	Preference for verbal information from healthcare provider	Information should be continuously accessible
Should be tailored to individual patients		Written information to consult at home	Information should be comprehensive
Relevant information topics: side effects, reason for use, working mechanism, therapy duration, treatment options, interactions, contra indications, use instructions			Information should come from a reliable source
			Information should be provided in an understandable manner

#### Theme I: Content of medication information

Patients were initially generally satisfied with the medication information that was provided. When discussed in more depth, almost all patients acknowledged to receive insufficient information that did not meet their needs. Patients emphasized that medication information needs were personal, and that information should be tailored to individual needs:

I think it is all so general. I would rather choose it to be different for each individual. I think it should be tailored to the person. (Rheumatology, male, 66 years)

What you want to know and don’t want to know is personal. There are people that think I have the information folder, but I do not read it and just let things happen. (Oncology, female, age unknown)

Patients expressed a need for various information topics about their medication, including side effects, reason for medication use, mechanism of action, length of therapy duration, (alternative) treatment options, interactions, contraindications, and medication use instructions. Informational needs about side effects varied between patients. For some patients, information about side effects was fearsome and they preferred not to be informed thereof because it would hinder them from taking their medication:

Yes, it is reality because also the one in ten thousand rare side effects are described. If you read all these, you will think, I never take these tablets again. (Rheumatology, female, 57 years)

#### Theme II: Moment of delivery

Patients were dissatisfied with the quantity and timing of information provided. Often an overload of information was given at start of therapy, when patients felt emotionally overwhelmed by the diagnosis. At this stage, patients highly trusted their physician and were less interested in and open to medication related information:

I was so occupied with my rheumatism. I only knew for ten days what was going on with me. Completely bedridden, completely depended. And then you get an information folder. My head was full, you cannot do that. (Rheumatology, female, 49 years)

#### Theme III: Method of delivery

Patients obtained medication information from a variety of sources. Patients highly preferred verbal information provided by the physician or nurse:

I really hate to say it, but I think that personal, direct contact works best. You have that with the rheumatologist, with the nurse, I think that is great. I can ask them everything. I will forget half of it, and then I ask again. I think that is, I am not into information from computers and folders. (Rheumatology, female, 59 years)

Some patients valued written information more, because verbal information could be easily forgotten, and written information could be consulted at home:

Preferably in writing because verbal, as soon as you get new medication, you already get so much information that, well, they say for sure things that they have told me before, but I just forget that because, in the first place I have heart failure. (Cardiology, male, 52 years)

#### Theme IV: Contextual quality prerequisites

Four quality requirements were identified that should be met according to patients’ needs: continuously accessible, comprehensive, reliable, and understandable. Medication information should be accessible during the complete course of therapy, because information needs could change over time:

What I liked about the information folder is that you can consult it. If you have some side effects, I think what should I do? I had some cold shivers and then I thought, I just read the folder, should I call the oncologist right now or can it wait until the next day. (Oncology, female, age unknown)

Patients indicated that the information provided should be comprehensive matching their personal needs:

I think it is difficult to say. I think that if you have a hundred people, you will get a hundred different stories. It remains always personal. I do think that patient informing is overlooked. I have experienced it myself when at the end of a consult I quickly received a folder without any verbal explanation. (Rheumatology, male, 68 years)

The rheumatologist is purely focusing on the medical aspect. And the other aspects are ignored, and, in my experience, too little medication information is given. You get al leaflet, but nothing is said about it. (Rheumatology, male, 68 years)

They specified a need for information from reliable sources, such as official websites and information brochures. Patients mentioned that not all people could judge the reliability of information sources and moreover, that they got anxious when reading stories online:

People are searching information on the internet and in general nothing is wrong with that, but if a person is not capable of understanding online medical information, then it can pose a real danger with getting the wrong information from unreliable websites. (Rheumatology, male, 68 years).

Patients remarked that medication information should be provided in a clear, short, and understandable manner. Especially information leaflets were perceived as less clear:

Information leaflets are often long, a bit messy and a bit unclear. (Cardiology, male, 49 years)

Moreover, also healthcare providers within and between hospitals should be able to communicate clearly:

I once proposed a question to the physician who said, yes, I can tell you because you understand it. You should be able to explain it to everyone. Yes, I then get very outrageous because I find that so arrogant. An intellectual person should be able to explain it to everyone. (Rheumatology, female, 70 years)

It was suggested that images and audio tapes could be used to increase understanding about medication:

Images work better than texts. I think it removes the language barrier. (Rheumatology, male 68 years)

### Quantitative phase

In total, 352 patients (cardiology n = 119, oncology n = 56, rheumatology n = 177), mean [SD] age 61.0 [13.1] years, 47.7% male, completed the questionnaire (Table 2).

**Table 2:** Characteristics of survey participants

	Cardiology (n = 119, %)			Rheumatology (n = 177, %)		Oncology (n = 56, %)	
Gender, male	72 (65.5)			71 (40.8)		25 (44.6)	
Mean [SD] age	65.3 [12.3]			57.0 [13.3]		64.3 [9.8]	
Disease type^a^	Heart rhythm disorder	54 (45.4)		Rheumatoid arthritis	117 (68.0)	Breast cancer	15 (26.8)
	Myocardial infarction	37 (31.4)		Psoriatic arthritis	30 (17.4)	Hematologic cancer	11 (19.6)
	Heart failure	36 (30.3)		Ankylosing spondylitis	20 (11.6)	Kidney cancer	9 (16.1)
	Angina pectoris	28 (23.5)		Other	13 (7.3)	Gastrointestinal cancer	7 (12.5)
	Hypertension	16 (13.4)				Skin cancer	6 (10.7)
	Congenital heart disease	13 (10.9)				Prostate cancer	4 (7.1)
	Other	15 (12.6)				Lung cancer	3 (5.4)
						Other	3 (5.4)

^a^Some patients reported multiple diseases and therefore the sum exceeds 100%

#### Current medication information

**Fig. 1:**
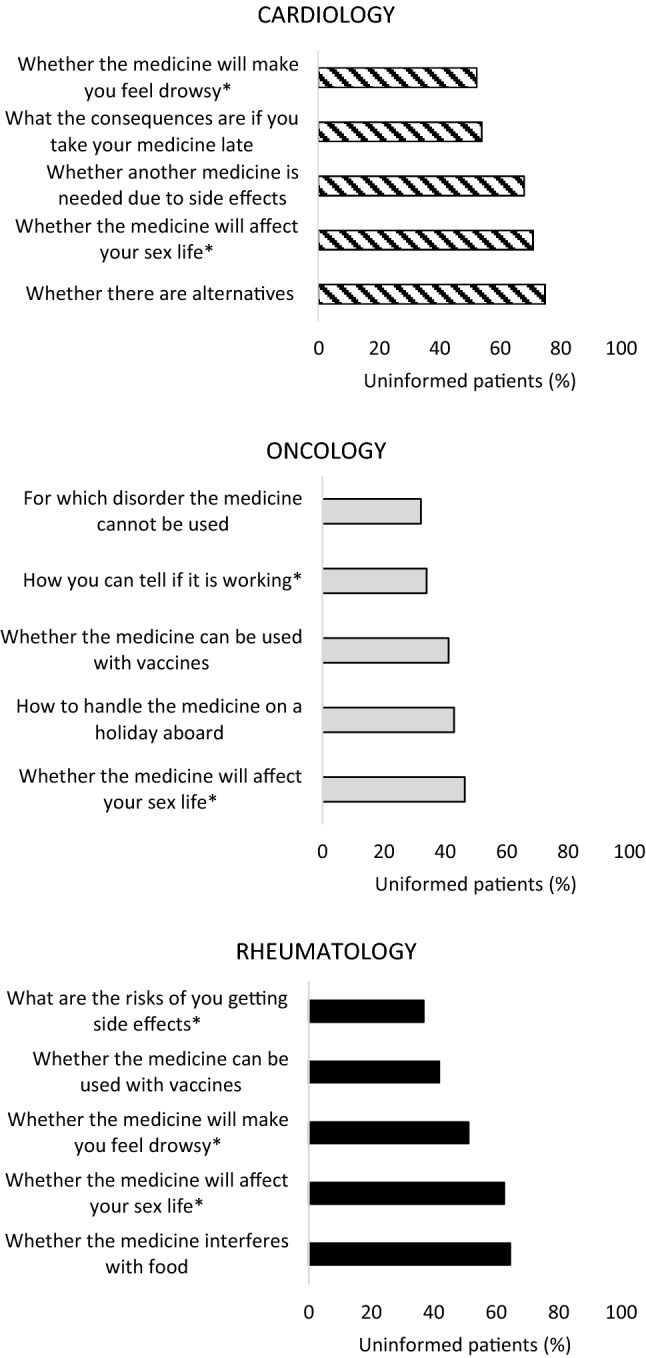
Top five items that patients were least informed about, with proportion of patients being uninformed. *Item corresponds with Satisfaction about Information Medicines Scale

Up to 74.6% of cardiology, 46.6% of oncology, and 62.6% of rheumatology patients were not informed about an item. Figure 1 shows the top five items that cardiology, oncology, and rheumatology patients were least informed about, which included amongst others “whether another medicine is needed due to side effects”, “whether the medicine will affect your sex life”, and “whether the medicine can be used with vaccines”. For full overview of proportion of patients not being informed per item, see supplementary material.

#### Medication information needs

Almost all patients reported all items as important content of medication information (Supplementary material, median ranking 4 or 5). There were no differences between patients who were informed and those who were not informed about an item and the perceived importance of the information. In all cases, patients who were not informed indicated that they valued this information as important. There were no large differences between cardiology, oncology, and rheumatology patients.

Most patients (cardiology 79.5%, oncology 87.5%, rheumatology 85.1%) received medication information from their prescriber, followed by the community pharmacy (39.8% cardiology patients), the nurse (44.6% oncology patients) and the outpatient hospital pharmacy (63.8% rheumatology patients). This is comparable to patients needs for information sources, with almost all patients having a need to receive information from the prescriber, followed by the pharmacy for over two-third of patients.

#### Quality of information

Patients were questioned about the quality of the received information. Table 3 shows the proportion of patients that scored the quality of information ‘very bad’ to ‘moderate’. A substantial number of patients were dissatisfied with the quality requirements of the information sources (accessible, comprehensive, reliable, and understandable). Cardiology patients reported the lowest quality compared with oncology and rheumatology patients. Up to one third of patients reported that verbal information from a healthcare provider and medication information leaflets were not sufficiently accessible, comprehensive, reliable and understandable. Even more patients indicated that the quality of medication information folders from the hospital, manufacturer, and patient organisation were of low quality. Many patients that used the internet or had received information from another patient, were dissatisfied about the quality thereof.

**Table 3:** Proportion of patients per disease category who scored the quality prerequisites of information sources from ‘very bad’ to ‘moderate’ (insufficient) indicating room for improvement

	Accessible						Comprehensive						Reliable						Understandable				
	(% insufficient)						(% insufficient)						(% insufficient)						(% insufficient)				
	Cardiology		Oncology		Rheumatology		Cardiology		Oncology		Rheumatology		Cardiology	Oncology		Rheumatology		Cardiology			Oncology		Rheumatology
*Verbal information*																							
Physician	37.5	10.7		32.5		33.0		14.3		19.0		17.1		3.6	10.2		21.9			14.3		10.9	
Nurse	33.7	12.5		21.9		29.8		4.8		11.9		14.5		1.8	9.6		20.7			5.4		6.7	
Pharmacist	32.7	23.2		17.3		38.3		32.7		12.9		23.0		21.4	9.9		33.3			21.4		8.7	
*Medication information leaflet*																							
Manufacturer	41.9	14.3		31.9		27.7		12.5		18.7		34.7		16.1	29.8		31.4			23.2		24.9	
Pharmacy	35.6	17.9		21.5		31.4		14.3		17.2		24.4		16.1	16.1		27.5			19.6		13.8	
*Medication information folder*																							
Hospital	31.8	10.7		28.5		32.9		21.4		12.1		27.8		12.5	15.3		28.0			14.3		11.9	
Manufacturer	51.4	16.1		38.3		44.4		25.0		25.7		41.6		14.3	32.0		39.7			19.6		26.1	
Patient organisation	41.0	19.6		21.2		35.9		21.4		16.9		29.7		16.1	18.6		33.9			19.6		16.5	
*Other*																							
Internet	37.0	–		27.4		44.3		–		24.8		44.9		–	39.8		39.7			–		27.4	
Information from another patient	70.5	21.4		62.7		64.6		60.9		57.3		61.7		19.6	64.4		60.9			23.2		57.4	

This was reported by patients who had received information from the specific source, e.g. patients who had used the internet scored the quality thereof

## Discussion

Almost all patients valued all medication information as important to receive, whereas a substantial number of patients had not received the information. Many patients perceived the quality of medication information, which included accessibility, comprehensiveness, reliability, and understandability, from multiple sources as insufficient.

During the qualitative data collection, patients indicated to receive insufficient information that does not meet their personal needs. This was confirmed in the quantitative data collection that showed that patients considered almost all information items as important but were not informed thereof. It appears that patients consistently have unmet information needs, which can have direct implications for optimal pharmacotherapy [1–4]. In this study, patients indicated that medication information should be tailored to their individual needs. Tailoring information should occur with respect to the content that is provided, moment of delivery, and method of delivery. Provided medication information at multiple moments both verbally and in writing can help improve patients understanding and knowledge about medication leading to proper and safe medication use. However, providing tailored information is complex as patients’ needs differ over the course of treatment.

Results of this study are in line with others showing that patients consider a wide range of information items relevant [14]. Substantial number of patients missed medication information. This mismatch may be due to lack of insight into patient needs among healthcare providers, which makes it difficult to provide relevant information. Indeed, research has demonstrated that healthcare providers have different views on information that should be provided to patients [10]. Furthermore, this can be due to factors such as limited time and barriers perceived among healthcare providers to discuss intimate topics. To overcome this problem, the patient-provider communication could be improved.

New to this study was also the assessment of the quality requirements that patients particularly valued as essential to be fulfilled. A substantial number of patients were dissatisfied with the quality of the provided medication information. This concerned a lack of information, which may have demanded patients to seek for information themselves, information that was not continuously accessible, and complex terminology used that cannot be easily understood by patients. Providing high quality information can meet patients’ unmet needs and be beneficial for patients to improve their medication use.

## Strengths and limitations

Strengths include that we performed a multicentre study with patients visiting different outpatient departments and that we used both qualitative and quantitative research methods. This enabled an in-depth identification of patients’ views and quantification among a larger sample of patients. Some limitations should be acknowledged. We were unable to collect data on number of patients refusing to participate and reasons thereof. Despite numerous efforts, we were only able to conduct one focus group for oncology patients and thus no data saturation was achieved. However, patients reported no new items during the survey and therefore we assume that no information was missed. During the quantitative phase patients were asked about information that they had received but we were not able to assess which information was actually provided to patients. Patients were directly asked to fill in the questionnaire after their consult with a healthcare provider and in our view, this limited to chance for recall bias. Furthermore, data were collected cross-sectionally although studies have shown that information needs vary over time. This study was carried out in the Netherlands and how and which information is provided to patients and what they need likely differs between countries due to differences in healthcare systems and cultural influences. Therefore, generalisation of the study results should be viewed with caution.

## Conclusion

Patients attending Dutch outpatient clinics have needs for extensive medication information, which should be tailored to their individual needs. Patients often face unmet needs because they receive less information than what they consider important. Four main quality prerequisites regarding medication information should be met according to patients, which included assessible, comprehensive, reliable, an understandable. Patients perceive current medication information of unsatisfactorily quality.

## Electronic supplementary material

Below is the link to the electronic supplementary material.

Supplementary file1 (DOCX 29 kb)

Supplementary file2 (DOCX 112 kb)

Supplementary file3 (DOCX 128 kb)

Supplementary file4 (DOCX 123 kb)
