# Supplementary material for: Information needs and patient perceptions of the quality of medication information available in hospitals: a mixed method study
Source: Int J Clin Pharm. 2020 Aug 28;42(6):1396–404. doi: 10.1007/s11096-020-01125-x (PMC7603457; doi:10.1007/s11096-020-01125-x)
Supplement: Supplementary file 1 — Supplementary file1 (DOCX 29 kb) [file 11096_2020_1125_MOESM1_ESM.docx]

**lgemene vragen**

1. Wat is uw geslacht?

☐ Man

☐ Vrouw

☐ Anders

2. Wat is uw leeftijd?

___________

3. Wat is uw hoogst genoten opleiding?

☐ Basisonderwijs

☐ Vmbo, mbo1 of avo-onderbouw

☐ Havo, vwo, mbo 2, 3 of 4

☐ Hbo of wo

4. Welke cardiologische aandoening(en) heeft u? *Meerdere antwoorden mogelijk.*

☐ Hartfalen

☐ Hartritmestoornissen

☐ Angina pectoris (pijn op de borst of hartkramp)

☐ Aangeboren hartziekten

☐ Hypertensie

☐ Hartinfarct of hartaanval

☐ Overig: _____________________________________

5. Gebruikt u medicijnen op recept voor uw hart- en vaatziekten?

☐ Ja

☐ Nee

**Ervaring met geneesmiddelinformatie**

6. Van wie heeft u deze informatie over uw hart- en vaatziekten medicijnen gekregen? *Meerdere antwoorden mogelijk.*

☐ Ik heb zelf de informatie opgezocht

☐ Van mijn arts

☐ Van de apotheek in het ziekenhuis waar ik ook de cardioloog bezoekt

☐ Van mijn eigen lokale apotheek

☐ Van mijn verpleegkundige specialist cardiologie

☐ Van de fabrikant van mijn hart- en vaatziekte medicijnen

☐ Van een andere patiënt die hetzelfde medicijn gebruikt

☐ Overig: __________________________________

7. Over welke onderwerpen heeft u informatie ontvangen?

|  | **Ja** | **Nee** | **N.V.T** |
| --- | --- | --- | --- |
| Wat de naam van het medicijn is | ☐ | ☐ | ☐ |
| Waarvoor het medicijn is | ☐ | ☐ | ☐ |
| Wat het medicijn doet | ☐ | ☐ | ☐ |
| Wat de voordelen van het medicijn zijn | ☐ | ☐ | ☐ |
| Hoe het medicijn werkt | ☐ | ☐ | ☐ |
| Hoe lang het duurt voor het medicijn werkt | ☐ | ☐ | ☐ |
| Hoe ik merk of het medicijn werkt | ☐ | ☐ | ☐ |
| Hoe lang ik het medicijn moet gebruiken | ☐ | ☐ | ☐ |
| Hoe ik het medicijn moet gebruiken | ☐ | ☐ | ☐ |
| Wat ik moet doen als ik een dosis ben vergeten in te nemen | ☐ | ☐ | ☐ |
| Wat de gevolgen zijn als ik mijn medicijn later inneem | ☐ | ☐ | ☐ |
| Hoe ik mijn herhaalmedicatie krijgt | ☐ | ☐ | ☐ |
| Of er (nieuwe) alternatieve medicijnen zijn voor mij | ☐ | ☐ | ☐ |
| Of het medicijn bijwerkingen heeft | ☐ | ☐ | ☐ |
| Wat de risico’s zijn dat ik bijwerkingen krijg | ☐ | ☐ | ☐ |
| Wat ik moet doen als ik bijwerkingen krijg | ☐ | ☐ | ☐ |
| Of het medicijn mijn seksleven beïnvloedt | ☐ | ☐ | ☐ |
| Of het medicijn mij suf kan maken | ☐ | ☐ | ☐ |
| Of er een ander medicijn nodig is ten gevolge van bijwerking van mijn medicijn | ☐ | ☐ | ☐ |
|  | **Ja** | **Nee** | **N.V.T** |
| Of er wisselwerkingen zijn met andere medicijnen | ☐ | ☐ | ☐ |
| Of ik alcohol mag drinken terwijl ik dit medicijn slik | ☐ | ☐ | ☐ |

8. Wat vindt u van de volledigheid van de medicijninformatie van de onderstaande informatiebronnen?

|  | **Heel**  **slecht** | **Slecht** | **Matig** | **Goed** | **Heel**  **goed** |
| --- | --- | --- | --- | --- | --- |
| Mondelinge informatie van arts | ☐ | ☐ | ☐ | ☐ | ☐ |
| Mondelinge informatie van verpleegkundige specialist cardiologie | ☐ | ☐ | ☐ | ☐ | ☐ |
| Mondelinge informatie van apotheek | ☐ | ☐ | ☐ | ☐ | ☐ |
| Bijsluiter medicijn van de fabrikant | ☐ | ☐ | ☐ | ☐ | ☐ |
| Patiëntbijsluiter van de apotheek | ☐ | ☐ | ☐ | ☐ | ☐ |
| Medicijnfolder van het ziekenhuis | ☐ | ☐ | ☐ | ☐ | ☐ |
| Medicijnfolder van de fabrikant | ☐ | ☐ | ☐ | ☐ | ☐ |
| Folder/ website van de hartstichting | ☐ | ☐ | ☐ | ☐ | ☐ |
| Het internet | ☐ | ☐ | ☐ | ☐ | ☐ |
| Informatie van een andere patiënt | ☐ | ☐ | ☐ | ☐ | ☐ |
| Overig: | ☐ | ☐ | ☐ | ☐ | ☐ |

9. Wat vindt u van de begrijpelijkheid van de medicijninformatie van de onderstaande informatiebronnen?

|  | **Heel**  **slecht** | **Slecht** | **Matig** | **Goed** | **Heel**  **goed** |
| --- | --- | --- | --- | --- | --- |
| Mondelinge informatie van arts | ☐ | ☐ | ☐ | ☐ | ☐ |
| Mondelinge informatie van verpleegkundige specialist cardiologie | ☐ | ☐ | ☐ | ☐ | ☐ |
| Mondelinge informatie van apotheek | ☐ | ☐ | ☐ | ☐ | ☐ |
| Bijsluiter medicijn van de fabrikant | ☐ | ☐ | ☐ | ☐ | ☐ |
| Patiëntbijsluiter van de apotheek | ☐ | ☐ | ☐ | ☐ | ☐ |
| Medicijnfolder van het ziekenhuis | ☐ | ☐ | ☐ | ☐ | ☐ |
| Medicijnfolder van de fabrikant | ☐ | ☐ | ☐ | ☐ | ☐ |
| Folder/ website van de hartstichting | ☐ | ☐ | ☐ | ☐ | ☐ |
| Het internet | ☐ | ☐ | ☐ | ☐ | ☐ |
| Informatie van een andere patiënt | ☐ | ☐ | ☐ | ☐ | ☐ |
| Overig: | ☐ | ☐ | ☐ | ☐ | ☐ |

10. Wat vindt u van de betrouwbaarheid van de medicijninformatie van de onderstaande informatiebronnen?

|  | **Heel**  **slecht** | **Slecht** | **Matig** | **Goed** | **Heel**  **goed** |
| --- | --- | --- | --- | --- | --- |
| Mondelinge informatie van arts | ☐ | ☐ | ☐ | ☐ | ☐ |
| Mondelinge informatie van verpleegkundige specialist cardiologie | ☐ | ☐ | ☐ | ☐ | ☐ |
| Mondelinge informatie van apotheek | ☐ | ☐ | ☐ | ☐ | ☐ |
| Bijsluiter medicijn van de fabrikant | ☐ | ☐ | ☐ | ☐ | ☐ |
| Patiëntbijsluiter van de apotheek | ☐ | ☐ | ☐ | ☐ | ☐ |
| Medicijnfolder van het ziekenhuis | ☐ | ☐ | ☐ | ☐ | ☐ |
| Medicijnfolder van de fabrikant | ☐ | ☐ | ☐ | ☐ | ☐ |
| Folder/ website van de hartstichting | ☐ | ☐ | ☐ | ☐ | ☐ |
| Het internet | ☐ | ☐ | ☐ | ☐ | ☐ |
| Informatie van een andere patiënt | ☐ | ☐ | ☐ | ☐ | ☐ |
| Overig: | ☐ | ☐ | ☐ | ☐ | ☐ |

11. Wat vindt u van de hoeveelheid medicijninformatie van de onderstaande informatiebronnen?

|  | **Heel**  **slecht** | **Slecht** | **Matig** | **Goed** | **Heel**  **goed** |
| --- | --- | --- | --- | --- | --- |
| Mondelinge informatie van arts | ☐ | ☐ | ☐ | ☐ | ☐ |
| Mondelinge informatie van verpleegkundige specialist cardiologie | ☐ | ☐ | ☐ | ☐ | ☐ |
| Mondelinge informatie van apotheek | ☐ | ☐ | ☐ | ☐ | ☐ |
| Bijsluiter medicijn van de fabrikant | ☐ | ☐ | ☐ | ☐ | ☐ |
| Patiëntbijsluiter van de apotheek | ☐ | ☐ | ☐ | ☐ | ☐ |
| Medicijnfolder van het ziekenhuis | ☐ | ☐ | ☐ | ☐ | ☐ |
| Medicijnfolder van de fabrikant | ☐ | ☐ | ☐ | ☐ | ☐ |
| Folder/ website van de hartstichting | ☐ | ☐ | ☐ | ☐ | ☐ |
| Het internet | ☐ | ☐ | ☐ | ☐ | ☐ |
| Informatie van een andere patiënt | ☐ | ☐ | ☐ | ☐ | ☐ |
| Overig: | ☐ | ☐ | ☐ | ☐ | ☐ |

12. Als u thuis informatie over uw hart- en vaatziekten medicijnen nodig heeft, hoe makkelijk kunt u dan de gewenste informatie uit de onderstaande informatiebronnen halen?

|  | **Heel**  **slecht** | **Slecht** | **Matig** | **Goed** | **Heel**  **goed** |
| --- | --- | --- | --- | --- | --- |
| Mondelinge informatie van arts | ☐ | ☐ | ☐ | ☐ | ☐ |
| Mondelinge informatie van verpleegkundige specialist cardiologie | ☐ | ☐ | ☐ | ☐ | ☐ |
| Mondelinge informatie van apotheek | ☐ | ☐ | ☐ | ☐ | ☐ |
| Bijsluiter medicijn van de fabrikant | ☐ | ☐ | ☐ | ☐ | ☐ |
| Patiëntbijsluiter van de apotheek | ☐ | ☐ | ☐ | ☐ | ☐ |
| Medicijnfolder van het ziekenhuis | ☐ | ☐ | ☐ | ☐ | ☐ |
| Medicijnfolder van de fabrikant | ☐ | ☐ | ☐ | ☐ | ☐ |
| Folder/ website van de hartstichting | ☐ | ☐ | ☐ | ☐ | ☐ |
| Het internet | ☐ | ☐ | ☐ | ☐ | ☐ |
| Informatie van een andere patiënt | ☐ | ☐ | ☐ | ☐ | ☐ |
| Overig: | ☐ | ☐ | ☐ | ☐ | ☐ |

**Behoefte aan informatie over uw hart- en vaatziekten medicijn**

13. Hoe vaak zou u gebruik maken van de onderstaande informatiebronnen als deze voor u beschikbaar zouden zijn?

|  | **Nooit** | **Zelden** | **Soms** | **Vaak** | **Altijd** |
| --- | --- | --- | --- | --- | --- |
| Mondelinge uitleg door zorgverlener | ☐ | ☐ | ☐ | ☐ | ☐ |
| Website | ☐ | ☐ | ☐ | ☐ | ☐ |
| Folder of brochure | ☐ | ☐ | ☐ | ☐ | ☐ |
| Bijsluiter | ☐ | ☐ | ☐ | ☐ | ☐ |
| Instructiefilmpje | ☐ | ☐ | ☐ | ☐ | ☐ |
| Overig: | ☐ | ☐ | ☐ | ☐ | ☐ |

14. Van wie zou u de informatie over uw hart- en vaatziekte medicijn willen ontvangen? *Meerdere antwoorden mogelijk.*

☐ Van mijn arts

☐ Van de apotheek in het ziekenhuis waar ik ook de cardioloog bezoek

☐ Van mijn eigen lokale apotheek

☐ Van mijn verpleegkundige specialist cardiologie

☐ Van de fabrikant van mijn hart- en vaatziekte medicijn

☐ Van een patiënt die hetzelfde hart- en vaatziekte medicijn gebruikt

☐ Overig: ___________________________________

15. Wanneer zou u de informatie over uw hart- en vaatziekte medicijn willen ontvangen? *Meerdere antwoorden mogelijk.*

☐ Voorafgaand aan de behandeling met het hart- en vaatziekte medicijn

☐ Tijdens de behandeling met het hart- en vaatziekte medicijn

☐ Na de behandeling met het hart- en vaatziekte medicijn

☐ Overig: ___________________________________

16. Hoe belangrijk vindt u het dat de volgende onderwerpen worden vermeld in de informatie over uw hart- en vaatziekte medicijn?

|  | **Onbelangrijk** | **Een beetje onbelangrijk** | **Neutraal** | **Een beetje**  **belangrijk** | **Belangrijk** |
| --- | --- | --- | --- | --- | --- |
| Wat de naam van het medicijn is | ☐ | ☐ | ☐ | ☐ | ☐ |
| Waarvoor het medicijn is | ☐ | ☐ | ☐ | ☐ | ☐ |
| Wat het medicijn doet | ☐ | ☐ | ☐ | ☐ | ☐ |
| Wat de voordelen van het medicijn zijn | ☐ | ☐ | ☐ | ☐ | ☐ |
| Hoe het medicijn werkt | ☐ | ☐ | ☐ | ☐ | ☐ |
| Hoe lang het duurt voor het medicijn werkt | ☐ | ☐ | ☐ | ☐ | ☐ |
| Hoe ik merk of het medicijn werkt | ☐ | ☐ | ☐ | ☐ | ☐ |
| Hoe lang ik het medicijn moet gebruiken | ☐ | ☐ | ☐ | ☐ | ☐ |
| Hoe ik het medicijn moet gebruiken | ☐ | ☐ | ☐ | ☐ | ☐ |
| Wat ik moet doen als ik een dosis ben vergeten in te nemen | ☐ | ☐ | ☐ | ☐ | ☐ |
| Wat de gevolgen zijn als ik mijn medicijn later inneem | ☐ | ☐ | ☐ | ☐ | ☐ |
| Hoe ik mijn herhaalmedicatie krijgt | ☐ | ☐ | ☐ | ☐ | ☐ |
| Of er (nieuwe) alternatieve medicijnen zijn voor mij | ☐ | ☐ | ☐ | ☐ | ☐ |
| Of het medicijn bijwerkingen heeft | ☐ | ☐ | ☐ | ☐ | ☐ |
| Wat de risico’s zijn dat ik bijwerkingen krijg | ☐ | ☐ | ☐ | ☐ | ☐ |
| Wat ik moet doen als ik bijwerkingen krijg | ☐ | ☐ | ☐ | ☐ | ☐ |
| Of het medicijn mijn seksleven beïnvloedt | ☐ | ☐ | ☐ | ☐ | ☐ |
| Of het medicijn mij suf kan maken | ☐ | ☐ | ☐ | ☐ | ☐ |
|  | **Onbelangrijk** | **Een beetje onbelangrijk** | **Neutraal** | **Een beetje**  **belangrijk** | **Belangrijk** |
| Of er een ander medicijn nodig is ten gevolge van bijwerking van mijn medicijn | ☐ | ☐ | ☐ | ☐ | ☐ |
| Of er wisselwerkingen zijn met andere medicijnen | ☐ | ☐ | ☐ | ☐ | ☐ |
| Of ik alcohol mag drinken terwijl ik dit medicijn slik | ☐ | ☐ | ☐ | ☐ | ☐ |

17. Zou u van de onderstaande stellingen aan kunnen geven in welke mate u het er mee eens bent met deze stelling:

|  | **Helemaal mee oneens** | **Oneens** | **Neutraal** | **Eens** | **Helemaal mee eens** |
| --- | --- | --- | --- | --- | --- |
| Ik vind dat er te veel informatie in één keer wordt gegeven | ☐ | ☐ | ☐ | ☐ | ☐ |
| Ik vind dat de informatie moet worden aangepast naar mijn eigen wensen | ☐ | ☐ | ☐ | ☐ | ☐ |
| Ik vind dat ik de informatie alleen van toepassing op mijn situatie moet ontvangen | ☐ | ☐ | ☐ | ☐ | ☐ |
| Ik vind dat er meer plaatjes gebruikt moeten worden | ☐ | ☐ | ☐ | ☐ | ☐ |
| Ik vind dat mijn arts voldoende kennis heeft | ☐ | ☐ | ☐ | ☐ | ☐ |
| Ik vind dat zorgverleners de voorlichting goed geregeld hebben | ☐ | ☐ | ☐ | ☐ | ☐ |
| Ik vind dat patiënten medicijninformatie zowel mondeling, schriftelijk als digitaal moeten kunnen ontvangen | ☐ | ☐ | ☐ | ☐ | ☐ |
| Ik vind dat patiënten medicijninformatie alleen mondeling moeten ontvangen | ☐ | ☐ | ☐ | ☐ | ☐ |
|  | **Helemaal mee oneens** | **Oneens** | **Neutraal** | **Eens** | **Helemaal mee eens** |
| Ik vind dat de medicijninformatie van verschillende zorgverleners met elkaar overeenkomt | ☐ | ☐ | ☐ | ☐ | ☐ |

18. Als u nog vragen of opmerkingen heeft naar aanleiding van deze vragenlijst dan kunt u die hieronder kwijt.

**Bedankt voor het invullen van de vragenlijst.**
