# Supplementary material for: Information needs and patient perceptions of the quality of medication information available in hospitals: a mixed method study
Source: Int J Clin Pharm. 2020 Aug 28;42(6):1396–404. doi: 10.1007/s11096-020-01125-x (PMC7603457; doi:10.1007/s11096-020-01125-x)
Supplement: Supplementary file 3 — Supplementary file3 (DOCX 128 kb) [file 11096_2020_1125_MOESM3_ESM.docx]

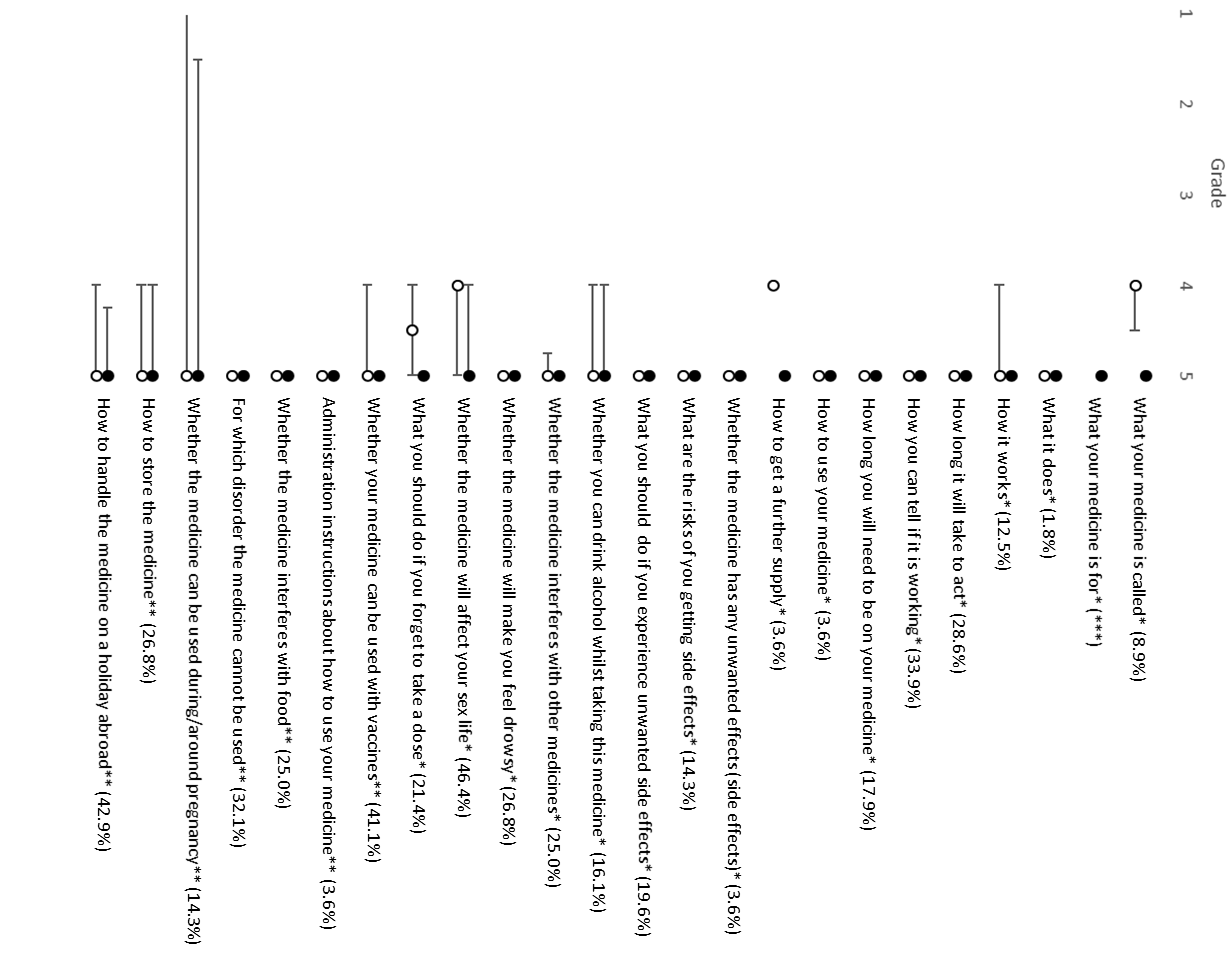


**Figure 2:** Median importance score with upper and lower quartile for each medication information item of oncology patients (1=unimportant to 5=important). In black score of informed patients, in white score of uninformed patients. Proportions represent the percentage of uninformed patients.

*Items corresponds with Satisfaction about Information Medicines Scale

**Disease specific items

***No patients were uninformed
